# Generation Y in der Chirurgie – der Konkurrenzkampf um Talente in Zeiten des Nachwuchsmangels

**DOI:** 10.1007/s00104-020-01138-2

**Published:** 2020-02-14

**Authors:** Kristian Nikolaus Schneider, Max Masthoff, Georg Gosheger, Nikolas Schopow, Jan Christoph Theil, Bernhard Marschall, Jürgen Zehrfeld

**Affiliations:** 1 Klinik für Allgemeine Orthopädie und Tumororthopädie, Universitätsklinikum Münster, Albert-Schweitzer Campus 1, 48149 Münster, Deutschland; 2 Institut für Klinische Radiologie, Universitätsklinikum Münster, Münster, Deutschland; 3 Klinik und Poliklinik für Orthopädie, Unfallchirurgie und Plastische Chirurgie, Universitätsklinikum Leipzig, Leipzig, Deutschland; 4 Institut für Ausbildung und Studienangelegenheiten, Universität Münster, Münster, Deutschland; 5 WWU Weiterbildung, Universität Münster, Münster, Deutschland

**Keywords:** Generation Y, Chirurgie, Studentische Ausbildung, Operationsassistenz, Nachwuchsförderung, Generation Y, Surgery, Student education, Operating room assistance, Promoting young talent

## Abstract

**Hintergrund:**

Chirurgische Fachdisziplinen kämpfen mit einem kritischen und sich zuspitzenden Nachwuchsproblem. Potenzielle Berufsanfänger zählen zur Generation Y, die Chefärzte und Personalabteilungen regelmäßig vor große Herausforderungen stellt. Ziel dieser Arbeit ist die Analyse verschiedener Maßnahmen der Personalakquise unter Berücksichtigung erhobener Motivationsfaktoren junger Medizinstudenten.

**Material und Methoden:**

Umfrage unter Medizinstudenten des 1. und 9. Fachsemesters (FS) einer medizinischen Fakultät zu individuellen Motivationsfaktoren, der angestrebten Facharztweiterbildung und der gesammelten Berufserfahrung in der Chirurgie.

**Ergebnisse:**

Ergebnisse von 179 der 269 befragten Medizinstudenten (66,5 %) konnten ausgewertet werden. Das Interesse an einer chirurgischen Facharztweiterbildung ist im 1. FS hoch (21 %) – fällt jedoch bis zum 9. FS deutlich ab (13 %; *p* = 0,23). Medizinstudenten, die im 9. FS „Aufstieg und Anerkennung“ gegenüber „flexible Arbeitszeiten“ präferieren, zeigen ein signifikant höheres Interesse an einer chirurgischen Weiterbildung (*p* = 0,022). Erworbene chirurgische Berufserfahrung wird mit einer durchschnittlichen Schulnote von 2+ bewertet.

**Schlussfolgerung:**

Das hohe Grundinteresse an einer chirurgischen Facharztweiterbildung zu Studienbeginn ist ein Wettbewerbsvorteil der Chirurgie. Die vielfältigen Rekrutierungsanstrengungen setzen jedoch oft erst gegen Ende des Studiums an. Zur langfristigen Nachwuchsbindung haben sich insbesondere frühzeitige Programme mit „Hands-on“-Charakter im chirurgischen Kernarbeitsbereich – dem Operationssaal – als erfolgreich erwiesen.

## Abstract (en)

**Background:**

Surgical disciplines are fighting with a critical and escalating shortage of recruits. Potential young professionals belong to the Generation Y, a generation that is constantly challenging senior consultants and human resources departments. The aim of this study was the analysis of various measures of personnel acquisition with respect to motivating factors of young medical students.

**Material and methods:**

A survey was carried out among students of the first and ninth semesters of a medical faculty on individual motivating factors, aspiration for medical specialist training and professional experience gained in surgery.

**Results:**

Results from 179 out of 269 medical students were available for analysis (66.5% response rate). The interest in a specialist training in surgery was high in the first semester of medical school (21%) but dropped noticeably up to the ninth semester (13%, *p* = 0.23). Medical students in the ninth semester, who favored professional advancement and appreciation over flexible working hours showed a significantly higher interest in a specialist training in surgery (*p* = 0.022). Surgical experience gained was valued with an average grade of 2+ (1 = best, 6 = worst).

**Conclusion:**

The high fundamental interest in a surgical residency during the beginning of medical studies is a competitive advantage of surgical disciplines; however, the diverse recruiting efforts are mainly aimed at later stages of studies. Timely hands-on courses in the core working area of surgery, the operating theatre, have proven to be particularly successful for the long-term acquisition and retention of junior doctors.

## Hintergrund und Fragestellung

Die chirurgische Versorgung leidet unter einem großen Personalbedarf, der sich einerseits aus einer Überalterung aktuell tätiger Chirurgen (dem sog. Ersatzbedarf), andererseits aber auch aus der demographisch bedingten Steigerung der Patientenzahlen (dem sog. Mehrbedarf) zusammensetzt.

Aktuell sind 30 % der ambulant und 11 % der stationär tätigen Chirurgen 60 Jahre oder älter und nur rund 2 % (ambulant) bzw. 21 % (stationär) 40 Jahre oder jünger [23, 24]. Gleichzeitig sind die stationären chirurgischen Behandlungsfälle in den vergangenen Jahren von 3.565.377 (2005) um knapp 12 % auf 3.992.185 (2016) gestiegen [25].

Der zunehmenden Überalterung der chirurgischen Ärzteschaft und den steigenden chirurgischen Fallzahlen steht nur ein geringes Interesse potenzieller Berufsanfänger gegenüber: Obwohl die Begeisterung für das Fach Chirurgie in den ersten Semestern noch hoch ist (40–60 % der Medizinstudenten können sich in den ersten Semestern eine chirurgische Berufslaufbahn vorstellen), hat nach Abschluss des praktischen Jahres nur noch eine einstellige Prozentzahl von Medizinstudenten Interesse an einer chirurgischen Facharztweiterbildung [16].

Viele chirurgische Kliniken in Deutschland leiden so unter einem massiven Bewerbermangel. In einer aktuellen Studie mit dem Titel „Nehmen wir jetzt jeden? – eine Umfrage in deutschen chirurgischen Kliniken“ geben 80 % aller Umfrageteilnehmer (715 chirurgische Chefärzte und [leitende] Oberärzte) an, einen *numerischen *Bewerbermangel wahrzunehmen. 94 % geben sogar an, einen *qualitativen *Mangel zu bemerken, sodass 90 % ihre Einstellungskriterien bereits herabgesenkt haben. 47 % freuen sich, „überhaupt Bewerbungen zu bekommen“, und 27 % geben an, aufgrund fehlender harter Ausschlusskriterien „jeden Bewerber einzustellen, der sich meldet“. Insgesamt 88 % erklären, dass der aktuelle Bewerbermangel sich negativ auf die Versorgungsqualität in chirurgischen Kliniken in Deutschland auswirkt [17].

Potenzielle Berufsanfänger zählen zur Generation Y (Geburtsjahrgänge ≈1981–2000), zu einer Generation die hohe Ansprüche an Personalabteilungen und Chefärzte stellt und von diesen erwartet, dass ihren Bedürfnissen auch nachgekommen wird [4–6, 8, 9]. Die jungen Ärzte der Generation Y bilden dabei eine besondere Kohorte, da sie – konträr zu ihren Vorgängergenerationen – bei ihrer Stellensuche von einem Fachkräftemangel im deutschen Gesundheitswesen profitieren: Während Chefärzte und Personalabteilungen in Zeiten der Ärzteschwemme (ca. 1982–2002) aus einem großen Kreis von Bewerbern auswählen und dabei die Arbeitsbedingungen vorgeben konnten, stellen nun Berufsanfänger ihrem zukünftigen Arbeitgeber Forderungen hinsichtlich der zu erfüllenden Arbeitsplatzbedingungen.

Studentische Fortbildungsveranstaltungen, „summer schools“ oder Mentoring- und Stipendienprogramme – die Rekrutierungsanstrengungen der chirurgischen Fachgesellschaften und Klinikleitungen sind vielfältig, jedoch bislang ohne durchschlagenden Erfolg: Die Anzahl chirurgisch interessierter Berufsanfänger bleibt trotz großer Begeisterung zu Studienanfang konstant niedrig [12, 16].

Ziel dieser Arbeit ist es, die primäre Begeisterung sowie den folgenden Interessenverlust an der Chirurgie während des Medizinstudiums zu erfassen und gezielt persönliche Interessengewichtungen und Motivationsfaktoren von Medizinstudenten unterschiedlicher Fachsemester zu erheben. Mithilfe der gewonnenen Erkenntnisse werden anschließend verschiedene Maßnahmen der Personalakquise diskutiert.

## Material und Methoden

Mithilfe einer anonymen Onlineumfrage unter Medizinstudenten des 1. und 9. Fachsemesters der medizinischen Fakultät der Westfälischen Wilhelms-Universität Münster wurden in sieben Einfachauswahlfragen zunächst individuelle Entscheidung in den Themengebieten „persönliche Entwicklung“, „Arbeitszeiten“, „Führung“, „Befindlichkeiten“, „berufliche Planbarkeit“, „Entfaltungsmöglichkeiten“, „Fehlerkultur“ und „Vergütung“ erfasst. Anschließend wurde in einem weiteren Abschnitt der Umfrage der Wunsch des späteren Weiterbildungsfaches sowie das Interesse an einer chirurgischen Weiterbildung auf einer Ratingskala und vorhandene Erfahrung in chirurgischen Fachdisziplinen per Freitexteingabe erhoben. Der freiwillige Fragebogen wurde im Rahmen der verpflichtenden Onlinesemesterabschlussevaluation im Sommersemester 2019 geschaltet.

Die statistische Auswertung der Daten erfolgte anschließend per SPSS Statistics 25 (IBM Corporation, Armonk/USA). Bei fehlender Normalverteilung der Daten wurden die Mediane verwendet und der Mann-Whitney-U-Test für nichtparametrische, unabhängige Stichproben zur Tendenz- und Signifikanztestung eingesetzt. Kategoriale Variablen wurden mittels χ^2^-Test verglichen. Das Signifikanzniveau wurde bei *p* < 0,05 festgesetzt, alle *p*-Werte waren zweiseitig.

## Ergebnisse

Insgesamt 179 von 269 Medizinstudenten (66,5 %) haben an der Umfrage teilgenommen, sodass Antworten von 101 der 150 Medizinstudenten (67 %) des 1. Fachsemesters sowie 78 der 119 Medizinstudenten (66 %) des 9. Fachsemesters ausgewertet werden konnten.

Die Ergebnisse der Einzelfragen sowie deren statistische Korrelation mit dem numerischen Interesse an der Chirurgie auf einer Ratingskala von 1 (schwach) bis 9 (stark) finden sich in Tab. 1. Da vereinzelte Fragen nicht von allen Medizinstudenten beantwortet wurden, kommt es zu geringfügigen Abweichungen bei der Gesamtantwortzahl.

**Table 1:** 

*1. Was sind für Sie unabdingbare Randbedingungen bei der Berufswahl?*				
Flexible Arbeitszeiten		Aufstieg und Anerkennung		Signifikanzen
1. Fachsemester	52 (52 %)	1. Fachsemester	48 (48 %)	*p* = 0,451
9. Fachsemester	56 (72 %)	9. Fachsemester	22 (27 %)	***p*** **=** **0,022**
*2. Wo würden Sie sich am meisten wohlfühlen?*				
Starke Führungspersönlichkeit eines fairen Chefarztes		Ausgeprägter Teamgedanke innerhalb der Abteilung		Signifikanzen
1. Fachsemester	24 (24 %)	1. Fachsemester	74 (75 %)	*p* = 0,838
9. Fachsemester	9 (11 %)	9. Fachsemester	67 (88 %)	*p* = 0,913
*3. Welche Fehlerkultur wünschen Sie sich bei Ihrem Arbeitgeber?*				
Kleinliche Analyse und Beurteilung von Fehlern		Positiv zugewandte Fehlerkultur		Signifikanzen
1. Fachsemester	8 (8 %)	1. Fachsemester	91 (91 %)	*p* = 0,817
9. Fachsemester	4 (5 %)	9. Fachsemester	74 (94 %)	*p* = 0,222
*4. Welche persönlich-beruflichen Entfaltungsmöglichkeit wünschen Sie sich von Ihrem Arbeitgeber?*				
Sabbatical (1 Jahr) zur freien Verfügung		Forschungsaufenthalt (1 Jahr) im Ausland		Signifikanzen
1. Fachsemester	63 (65 %)	1. Fachsemester	33 (34 %)	*p* = 0,781
9. Fachsemester	57 (75 %)	9. Fachsemester	19 (25 %)	*p* = 0,382
*5. Sie sind von Ihrer Chefärztin als Veranstaltungsbeauftragte/r der Klinik benannt worden. Für das kommende Jahr gibt es noch erhebliche finanzielle Kapazitäten, die zwingend verplant werden müssen, da sie sonst verfallen. Ihre Chefärztin hat mehrfach betont, dass Sie volle Handlungs- und Entscheidungsgewalt besitzen und sie keinesfalls mit Planungs- oder Organisationsfragen belästigt werden möchte. Was organisieren Sie?*				
Herrliches Sommerfest für alle Mitarbeiter der Abteilung in einer Szene-Location der Stadt		Hochkarätiges Fachsymposium mit renommierten Referenten aus Pflege und Verwaltung für alle Mitarbeiter der Abteilung		Signifikanzen
1. Fachsemester	63 (65 %)	1. Fachsemester	33 (34 %)	*p* = 0,406
9. Fachsemester	55 (72 %)	9. Fachsemester	21 (27 %)	*p* = 0,924
*6. Sie haben das erste Assistenzarztjahr erfolgreich absolviert und treffen auf der angesagten Geburtstagsfeier eines Nachbarn (Freitagabend – 19 Uhr), der Sie besonders eingeladen hat, eine befreundete Operationsschwester, die Ihnen mitteilt, dass um 21 Uhr in der Klinik eine seltene, mehrstündige Operation ansteht, die fachlich genau in Ihr Interessengebiet fällt. Wie verhalten Sie sich?*				
Ich enttäusche meinen Nachbarn nicht und bleibe auf der Geburtstagsfeier		Ich bin um 21 Uhr im Operationssaal der Klinik		Signifikanzen
1. Fachsemester	43 (43 %)	1. Fachsemester	55 (56 %)	*p* = 0,587
9. Fachsemester	34 (44 %)	9. Fachsemester	42 (55 %)	*p* = 0,063
*7. Was wünschen Sie sich eher von Ihrem Arbeitgeber?*				
Übernahme von Fort- und Weiterbildungskosten		Finanzielle Beteiligung analog zum eigenen Beitrag am Klinikerfolg		Signifikanzen
1. Fachsemester	69 (69 %)	1. Fachsemester	30 (30 %)	*p* = 0,884
9. Fachsemester	68 (89 %)	9. Fachsemester	8 (10 %)	*p* = 0,404

Es fällt auf, dass im Studienverlauf „Aufstieg und Anerkennung“ (gegenüber „flexiblen Arbeitszeiten“; *p* = 0,005), eine „finanzielle Beteiligung analog zum eigenen Beitrag am Klinikerfolg“ (gegenüber der „Übernahme von Fort- und Weiterbildungskosten“; *p* = 0,007) sowie der einjährige Forschungsaufenthalt im Ausland (gegenüber dem „einjährigen Sabbatical zur freien Verfügung“; *p* = 0,002) signifikant an Bedeutung verlieren (s. Tab. 1).

**Figure 1:**
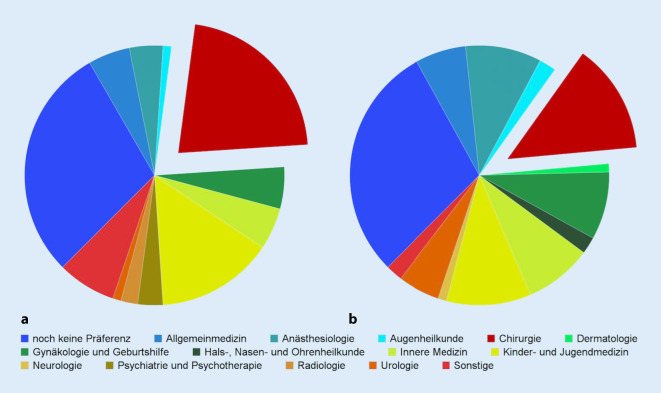

Hinsichtlich der Häufigkeitsverteilung führt die Chirurgie sowohl im 1. Fachsemester als auch im 9. Fachsemester die Statistik an – ihr Verlust im Laufe des Medizinstudiums ist allerdings deutlich, wenngleich nicht signifikant: von 21 % im 1. Fachsemester auf 13 % im 9. Fachsemester (*p* = 0,23; Abb. 1).

**Figure 2:**
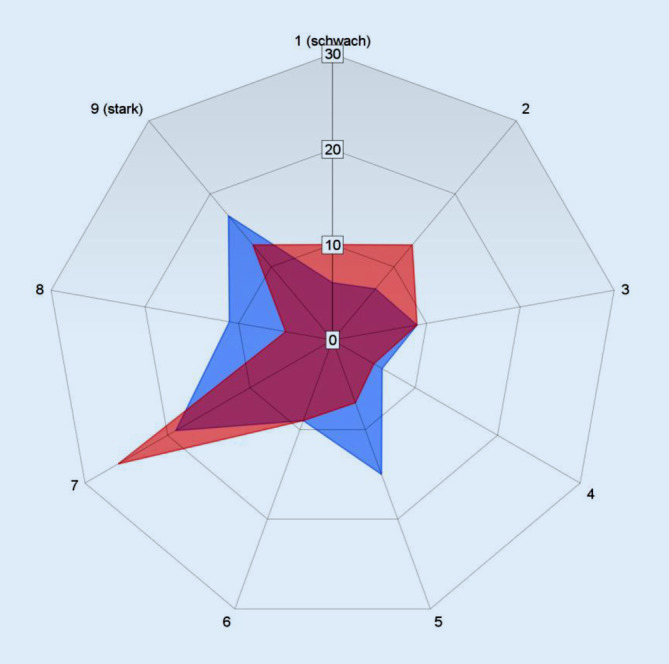

Medizinstudenten des 1. Fachsemesters können sich nicht nur häufiger eine chirurgische Weiterbildungsstelle vorstellen, ihr Interesse an der Chirurgie ist auch stärker ausgeprägt als im 9. Fachsemester: Bei identischen Medianen unterscheiden sich die jeweiligen Interquartilsabstände: 1. Fachsemester (4–8) und 9. Fachsemester (3–7), (*p* = 0,187; Abb. 2).

Für Medizinstudenten, die im 9. Fachsemester „Aufstieg und Anerkennung“ über „flexible Arbeitszeiten“ wählen, konnten wir ein signifikant höheres Interesse an einer chirurgischen Weiterbildung nachweisen als für die Medizinstudenten, die sich im 9. Fachsemester für „flexible Arbeitszeiten“ entscheiden würden (*p* = 0,022, s. Tab. 1).

Im 1. Fachsemester geben 22 % der Medizinstudenten an, bereits Berufserfahrung in der Chirurgie gesammelt zu haben, im 9. Fachsemester knapp 60 % der Medizinstudenten. Die gesammelten Erfahrungen werden dabei fast ausnahmslos mit guten bis sehr guten Schulnoten bewertet (⌀: 11,66 Punkte, durchschnittliche Schulnote ≈2+).

## Diskussion

Die wichtigsten Ergebnisse unserer Studie sind: (1.) Das Interesse an der Chirurgie nimmt im Studienverlauf ab. (2.) Während dieser Zeit ist eine teils signifikante Verschiebung einzelner Motivationsfaktoren zu beobachten. (3.) Im Rahmen der Nachwuchsakquise nutzen chirurgische Fachgesellschaften ihre Startvorteile und Alleinstellungsmerkmale gegenüber anderen Fachdisziplinen nicht ausreichend aus. Ein Modell zur Nachwuchsgewinnung, welches das hohe Grundinteresse an der Chirurgie sowie gezielt wichtige Motivationsfaktoren der Generation Y berücksichtigt, wird abschließend vorgestellt.

Während des Medizinstudiums ist ein viel zitierter, aber nur selten quantifizierter Interessenverlust an einer späteren chirurgischen Facharztweiterbildung zu beobachten. So berichten Osenberg et al. von 34 % der Medizinstudenten, die im 1. Semester ein Interesse an einer chirurgischen Weiterbildung haben, im letzten Semester seien dies jedoch nur noch 16 %. Interessanterweise lässt sich dieser Interessenverlust, den wir bestätigen konnten, nicht auf andere Länder übertragen: Während es in Deutschland einen deutlichen numerischen und qualitativen Bewerbermangel in chirurgischen Fachkliniken gibt, zeigt sich in den USA ein deutlicher Bewerberüberhang, der dazu führt, dass nicht alle Medizinstudenten ihre gewünschte chirurgische Facharztausbildung beginnen können [1, 2, 17].

Zahlreiche Autoren haben die Motivationsfaktoren, Interessengewichtungen, Wünsche und Erwartungshaltungen von Medizinstudenten und jungen Ärzten der Generation Y untersucht und mit denen der vorherigen Generationen verglichen [4]. Gemeinsames Fazit der Studien ist der hohe Stellenwert einer guten „Work-life-Balance“, des „fachlichen Anspruchs“, „angenehmen Betriebsklimas“ und „Prestiges des Arbeitsgebers“ – die „Work-life-Balance“ wird dabei sogar als „nicht verhandelbare, unabdingbare Arbeitsplatzbedingung“ eingeordnet [4]. Die Mehrheit der Generation Y zeichnet sich zudem durch ein gesteigertes Sicherheitsbedürfnis und eine hohe schulische Leistungsmotivation aus, die eine gute Ausgangslage auf dem Arbeitsmarkt ermöglichen soll [10].

In der öffentlichen Wahrnehmung sowie in der Tagespresse wird die Generation Y oftmals als „verlorene Generation“ abgestempelt, die nicht nur „überfordert und gierig“, sondern auch noch „faul und selbstüberschätzend“ sei [14, 18]. Diese Feststellungen lassen sich in unserer Befragung nur bedingt bestätigen.

Wir konnten zwar zeigen, dass Motivationsfaktoren wie „Aufstieg und Anerkennung“, „eine finanzielle Beteiligung analog zum eigenen Beitrag am Klinikerfolg“ sowie ein „einjähriger Forschungsaufenthalt im Ausland“ im Studienverlauf signifikant an Bedeutung verlieren, doch der Arbeitseifer und Karrieredrang der Generation Y ist nicht zu unterschätzen: 56 % (1. Fachsemester) bzw. 55 % (9. Fachsemester) der Medizinstudenten würden sich an einem Freitagabend um 19 Uhr für die freiwillige Teilnahme an einer außerplanmäßigen, mehrstündigen Operation aus dem eigenen Interessengebiet entscheiden, statt einer privaten Einladung zu folgen. Osenberg et al. postulieren, dass Medizinstudenten jedoch von der „hohen psychosozialen Arbeitsbelastung“ in der Chirurgie abgeschreckt werden und „nach einem intensiven und langen Studium weniger bereit sind, sich auch im weiterführenden Berufsleben auf Kompromisse und Entbehrungen einzustellen“ – hierzu passt auch der von uns im Studienverlauf beobachtete signifikante Bedeutungsverlust von „Aufstieg und Anerkennung“ gegenüber „flexibler Arbeitszeiten“ [12].

Personalabteilungen und Chefärzte haben auf die veränderten Interessengewichtungen der Nachwuchsärzte der Generation Y reagiert und unter dem Druck des zunehmenden Ärztemangels ihre Personalakquise und die Arbeitsbedingungen in den Kliniken der Situation angepasst.

Medizinstudenten werden dabei zielgerichtet und engmaschig nicht nur von möglichen späteren Arbeitgebern, sondern auch von verschiedenen Fachgesellschaften umworben. So ist ein „ungewollter Wettbewerb der Fächer in der Medizin“ entstanden, der kritisch betrachtet werden muss [13].

Fortbildungsveranstaltungen oder auch „summer schools“ sind eine weit verbreitete Möglichkeit zur Nachwuchsgewinnung und gleichzeitigen Imagepflege [3]. Unsere Umfrage bestätigt, dass die aktuelle Generation der Medizinstudenten einerseits eine hohe Bereitschaft zur Weiterbildung außerhalb der regulären Studien‑/Arbeitszeit aufweist, andererseits aber auch ein starkes Interesse an der Übernahme von Fort- und Weiterbildungskosten zeigt.

Da jedoch mittlerweile fast sämtliche Fachgesellschaften entsprechende Veranstaltungen anbieten, ist das ursprüngliche Alleinstellungsmerkmal einiger weniger innovativer Fachverbände bereits abgeschwächt [3]. Um dennoch weiter Medizinstudenten für das eigene Fach oder die eigene Klinik zu begeistern, wird über Stipendienangebote zunehmend auch monetär um interessierte Medizinstudenten geworben, die sich im Gegenzug oftmals zu vertraglichen Gegenleistungen verpflichten müssen [19, 20].

Dort, wo das Werben um Nachwuchsärzte auf klassischen Wegen nicht mehr funktioniert, wird es von staatlicher Seite durchgesetzt: Mit dem „Landarztgesetz“ hat das Land Nordrhein-Westfalen (NRW) im Jahre 2018 einen radikalen Weg der Akquise fehlender Hausärzte in ländlichen Regionen beschlossen: Pro Semester werden 170 Studienplätze der Humanmedizin (7,6 % aller Humanmedizinstudienplätze in NRW) an Bewerber vergeben, die sich vertraglich verpflichten, nach Abschluss des Studiums eine ärztliche Weiterbildung zu absolvieren, die (1.) Voraussetzung für eine Tätigkeit als Hausarzt ist und (2.) anschließend für zehn Jahre eine vertragsärztliche Tätigkeit dort auszuüben, wo ein besonderer Bedarf an Hausärzten besteht. Bei Verstoß gegen eine der beiden vertraglich festgelegten Verpflichtungen, droht eine Vertragsstrafe in Höhe von 250.000 € [21].

Bei der prekären und sich auch in anderen Fachdisziplinen zuspitzenden Nachwuchssituation scheint es nur eine Frage der Zeit, wann auch die ersten Rufe anderer Fachgesellschaften nach einer gesetzlichen Quote für ihre Fachdisziplin folgen werden. Im Extremfall könnte das Medizinstudium so zu einer Art Planstudium verkommen, in dem schon zu Studienbeginn die spätere Facharztweiterbildung der Erstsemester vertraglich bindend festgelegt werden und der natürliche Prozess, sich im Laufe des Studiums von einer Facharztweiterbildung begeistern zu lassen, übergangen wird.

Die Chirurgie hat gegenüber anderen Fachdisziplinen den Vorteil eines hohen Grundinteresses in frühen Studienabschnitten und, dass der Operationssaal neben der Sprechstunden- und Stationsarbeit einen weiteren wichtigen Tätigkeitsschwerpunkt ausmacht.

Doch beide Wettbewerbsvorteile verspielt die Chirurgie leichtfertig: Statt bei der Nachwuchsakquise schon im vorklinischen Bereich anzusetzen oder das Alleinstellungsmerkmal „Operationssaal“ einzusetzen, konzentrieren sich die Rekrutierungsanstrengungen chirurgischer Fachgesellschaften und Kliniken oftmals ausschließlich auf die späteren Studienabschnitt und insbesondere auf das Praktische Jahr [15, 22].

Gleichzeitig erhalten zudem wohl nur wenige Medizinstudenten einen adäquat frühzeitigen Einblick in die chirurgische Arbeit im Operationssaal und das, obwohl fast alle Studenten verpflichtende Praktika und auch ein obligatorisches Chirurgietertial im Rahmen des Praktischen Jahres (11. und 12. Fachsemester) absolvieren müssen. In unserer Umfrage haben knapp 40 % der Medizinstudenten des 9. Fachsemesters angegeben, noch keine Erfahrung in der Chirurgie gesammelt zu haben.

Dabei wird die gesammelte Berufserfahrung in chirurgischen Fächern generell gut bewertet: In einer Umfrage von Sutton et al. unter 482 Medizinstudenten konnte gezeigt werden, dass Praktika in chirurgischen Fachdisziplinen besser bewertet werden als in anderen Fächern [11]. Auch die Medizinstudenten in unserer Umfrage haben ihre gesammelten Erfahrungen generell gut bis sehr gut bewertet (durchschnittliche Schulnote ≈2+).

Eine interessante Option, Medizinstudenten ohne langfristige vertragliche Gegenleistungen für eine spätere Tätigkeit in einer chirurgischen Abteilung zu begeistern, ist die Möglichkeit, Medizinstudenten als studentische Hilfskraft (SHK) im Operationssaal einzusetzen.

Ein Modell, welches nicht nur in unserer Klinik erfolgreich etabliert wurde und bei dem chirurgisch interessierte Medizinstudenten ab dem 2. Fachsemester die Chance erhalten, exklusive Einblicke in komplexe operative orthopädische Eingriffe zu gewinnen und mit zunehmendem Erfahrungsschatz auch selbstständig kleinere Assistenzen im Operationssaal zu übernehmen. Die SHK werden dabei nicht nur im Operationssaal als vollwertige Teammitglieder gesehen, sondern auch in das gesellschaftliche Leben der Klinik integriert. Der ihnen entgegengebrachte Respekt überrascht viele SHK und spricht gezielt mehrere von der Generation Y als wichtig eingestufte Arbeitsplatzkriterien an: ein positives Arbeitsplatzklima, einen starken Teamgedanken sowie eine hohe Wertschätzung. So entsteht eine Arbeitsplatzatmosphäre, die viele SHK so begeistert, dass sie anschließend ihre Weiterbildung zum Facharzt für Orthopädie und Unfallchirurgie in unserer Klinik beginnen.

Ähnlich positive Erfahrungen haben schon andere Autoren mit vergleichbaren Modellen für chirurgische Rufdienst‑/Schockraumassistenzen in deutschen Universitätsklinika gemacht [7, 10]. Rabe et al. haben neben den beruflichen und finanziellen Vorteilen für die Medizinstudenten auch die Kostenersparnis des Arbeitgebers herausgearbeitet und betonen, dass es sich bei dem Modell um eine finanzielle und berufliche „Win-win-Situation“ für Medizinstudenten und chirurgische Klinik handelt [7].

Spering et al. konnten zeigen, dass sich 82 % der SHK anschließend auf orthopädische/unfallchirurgische Assistentenstellen beworben haben, alle Bewerber ihre Wunscharbeitsstelle erhielten und bei ihrem späteren Arbeitgeber durch eine „beschleunigte Orientierung im Operationssaal“ und eine „signifikant verkürzte Einarbeitungszeit“ bis zur ersten Dienstreife (SHK ⌀ 14 Tage, Jungassistent ⌀ 41 Tage) ausgezeichnet haben [10].

Selbstverständlich sind die beschriebenen Modelle leichter in Universitätskliniken oder in Kliniken mit einer räumlichen Nähe zu medizinischen Fakultäten zu implementieren. Für chirurgische Abteilungen ohne diese Gegebenheiten gilt die Empfehlung von Sutton et al., informelle Mentorenbeziehungen schon zu Schüler‑, Pflegepraktikanten, Hospitanten und Famulanten aufzubauen und diese über den Studienverlauf hin aufrechtzuerhalten, da aus jedem Schülerpraktikanten ein späterer chirurgischer Facharzt werden könnte [11].

Unsere Studie hat Schwachstellen: (1.) Es fehlt ein dritter Befragungszeitpunkt nach dem Praktischen Jahr. Für unsere Studie konnten wir auf die verpflichtende Semesterabschlussevaluation (Semester 1 und 9) der medizinischen Fakultät zurückgreifen, die trotz freiwilliger Beantwortung der Fragen eine vergleichsweise hohe Rücklaufquote von 67 % sichergestellt hat. Für das Praktische Jahr hätte eine zusätzliche Befragung per fakultativem Fragebogen erfolgen müssen. Dies hätte aufgrund der geringen Rücklaufquote anderer Autoren mit diesem Instrument (≈14–19 %; [2, 3]) zu einer Verzerrung der Ergebnisse und zu einer fehlenden Vergleichbarkeit geführt. (2.) Es wurden nur Medizinstudenten einer einzelnen Fakultät befragt. Eine Ausweitung auf andere Fakultäten ist ein guter Ansatz für Folgestudien, um Unterschiede in der studentischen Ausbildung und deren Auswirkungen auf das chirurgische Interesse der Medizinstudenten zu identifizieren und zu analysieren. (3.) Fehlendes Antwortverhalten von nichtchirurgisch interessierten Medizinstudenten könnte zu einem Nonresponsebias geführt haben. Auch wenn knapp 33 % der Medizinstudenten nicht an der Umfrage teilgenommen haben, haben wir mit dem Studiendesign unter Verwendung kurzer, prägnanter Fragen und unter Wahrung der absoluten Anonymität der Teilnehmer versucht, einem möglichen Bias entgegenzuwirken.

## Schlussfolgerung

Chirurgische Fachdisziplinen kämpfen mit einem kritischen und sich zuspitzenden Nachwuchsproblem. Fachgesellschaften, Kliniken, Personalabteilungen und Chefärzte sollten das ausgeprägte Grundinteresse der Studenten an der Chirurgie in frühen Semestern als Wettbewerbsvorteil nutzen und zielgerichtet das intrinsische Interesse von Medizinstudenten mittels interaktiver Programme und „Hands-on“-Charakter ansprechen. Neben Fortbildungsreihen, die die praktischen und manuellen Fähigkeiten der Medizinstudenten fördern, haben sich studentische Assistenzen im chirurgischen Kernarbeitsbereich – dem Operationssaal – als langfristig erfolgreich erwiesen.

## Fazit für die Praxis

- Chirurgische Fachdisziplinen kämpfen mit einem kritischen und sich zuspitzenden Personalbedarf.
- Im Studienverlauf ist ein signifikanter Bedeutungsverlust mehrerer extrinsischer Motivationsfaktoren zu beobachten.
- Chirurgische Berufserfahrung in Form von Schülerpraktika, Blockpraktika, Famulaturen oder studentischen Nebenjobs wird generell gut bis sehr gut bewertet (durchschnittliche Schulnote ≈2+), aber nur wenige Medizinstudenten können vor dem Praktischen Jahr ausreichende chirurgische Berufserfahrung in Form von Blockpraktika und Famulaturen sammeln.
- Die Rekrutierungsanstrengungen von chirurgischen Fachgesellschaften und Personalabteilungen sind vielfältig, setzen jedoch oft erst zum Studienende mit monetären, extrinsischen Anreizen an.
- Zur langfristigen Nachwuchsbindung haben sich insbesondere interaktive Programme mit „Hands-on“-Charakter, wie z. B. studentische Assistenzen im chirurgischen Kernarbeitsbereich – dem Operationssaal – als erfolgreich erwiesen.
